# Combined Rheumatic Mitral and Aortic Stenosis: Hemodynamics and a Tailored Approach to Management

**DOI:** 10.1016/j.jaccas.2025.104670

**Published:** 2025-08-20

**Authors:** Bhanu Duggal, Vijay Kumar Varada, Anirudh Mukherjee, Raghuraj Chawla, Rupendra Nath Saha

**Affiliations:** Department of Cardiology, AIIMS Rishikesh, Rishikesh, Uttarakhand, India

**Keywords:** aortic stenosis, balloon mitral valvotomy, double valve replacement, mitral stenosis, rheumatic heart disease

## Abstract

**Background:**

The greatest rates of rheumatic heart disease (RHD) are currently seen in South Asia, central sub-Saharan Africa, and Oceania. Mitral and aortic stenosis involvement is one of the rarest combinations, affecting only 2.4% of patients.

**Case Summary:**

We describe 3 patients with concomitant severe rheumatic aortic and mitral stenosis, each with a unique clinical presentation. Each case highlights the complex hemodynamic nature of the combined stenotic lesion.

**Discussion:**

Double valve replacement is usually recommended as the treatment of choice for these stenotic lesions. However, such a universal approach for all stenotic lesions is problematic owing to the complications that can occur during the perioperative and postoperative periods of double valve replacement.

**Take-Home Message:**

Simultaneous percutaneous transvenous mitral commissurotomy and aortic valve replacement can be a treatment strategy for concomitant severe rheumatic aortic and mitral stenosis, especially in low- and middle-income countries.

## Graphical Abstract

**Figure:**
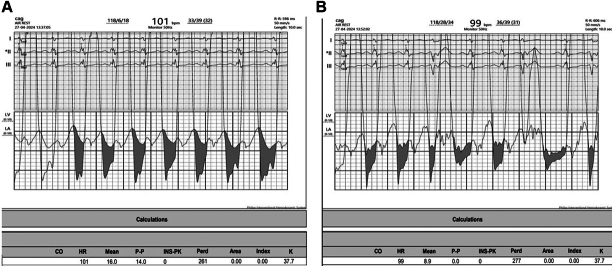

Global patterns of rheumatic heart disease (RHD) can be categorized as either endemic (high childhood mortality and prevalence) or nonendemic (low childhood mortality and prevalence, with predominance during older age). In 2015, countries with a nonendemic RHD pattern reported an incidence of 3.4 per 100,000 people; in contrast, countries with an endemic pattern reported an incidence of 444 per 100,000.^1^ The greatest rates of RHD are seen in South Asia, central sub-Saharan Africa, and Oceania. RHD predominantly affects multiple valves, and it can manifest in various presentations, including mixed regurgitant and stenotic lesions of the heart valves. Mitral and aortic stenosis is one of the rarest combinations, affecting only 2.4% of patients.^2^ Even the diagnosis and proper staging of such diseases are challenging, as most of the conventional echocardiographic techniques for assessing severe stenosis physiology, such as pressure half-time or a mean valvular gradient, are generally defined in isolated valvular diseases and thus may fall short when there are mixed valvular lesions.^3^ Here, we describe 3 cases of concomitant severe rheumatic aortic and mitral stenosis, each with a unique clinical presentation.

**Take-Home Messages**

• The severity of aortic lesions can be challenging to assess in a patient with preexisting severe mitral stenosis.
• Aortic regurgitation or anemia increase the flow rate across the aortic valve and may cause a false elevation in gradients, which should be considered carefully.
• An aortic valve area by planimetry is one of the most accurate measures of predicting aortic valve disease burden, but unfortunately, poor echo windows limit this mode of direct measurement.
• Simultaneous percutaneous transvenous mitral commissurotomy and aortic valve replacement can be a treatment strategy for concomitant severe rheumatic aortic and mitral stenosis, especially in low- and middle-income countries.

## Case 1

**Figure 1:**
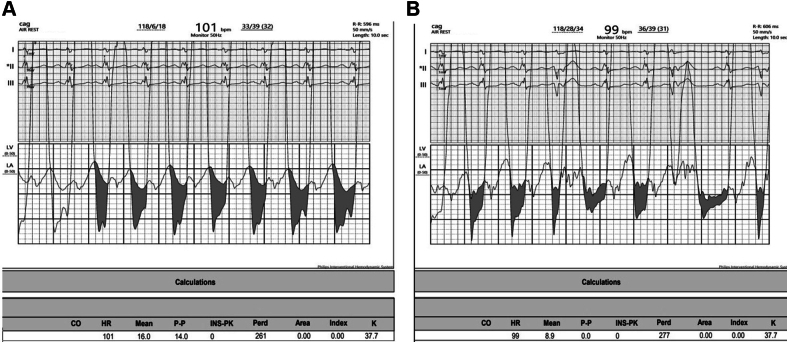
Hemodynamic Tracings Catheter tracings showing the gradient across the mitral valve (A) before and (B) after percutaneous transvenous mitral commissurotomy.

**Figure 2:**
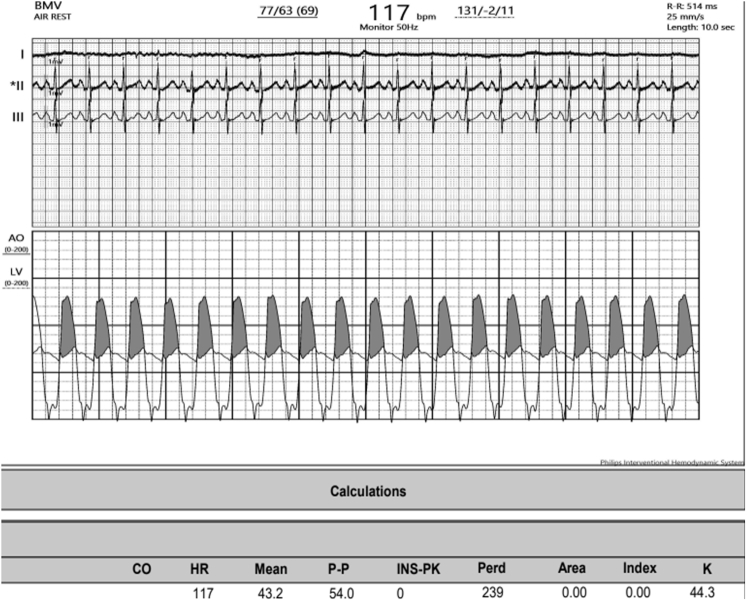
Hemodynamic Tracings Catheter tracings showing the simultaneous left ventricle–aorta gradient after percutaneous transvenous mitral commissurotomy.

A 27-year-old woman with 2 previous uneventful pregnancies presented with dyspnea on exertion that had lasted for 2 years, which had progressed over 6 months from NYHA functional class II to III was associated with intermittent episodes of nonexertional palpitations of sudden onset and offset for 6 months. Cardiac examination and 2-dimensional echocardiography were suggestive of severe mitral stenosis with severe aortic stenosis and moderate pulmonary hypertension (Video 1, Tables 1 and 2) After a discussion with the heart team, she underwent a successful percutaneous percutaneous transvenous mitral commissurotomy (Video 2, Figures 1 and 2, Table 3). Figure 1 show the simultaneous tracings of the left ventricle and left atrium, with the shaded portion showing the gradients between the 2 chambers, while Figure 2 shows the simultaneous gradients between the left ventricle and aorta, thus giving a real-time assessment of the gradients across the aortic valve.

**Table 1:** Symptoms Shown by the Patients

Patient	Symptoms	Apex	A2-OS Interval	Murmur in Apex	Murmur in Base	Rhythm	Additional Sounds	P2	Hemoglobin (g/dL)
Patient 1	DOE, NYHA functional class IV	Fifth IC, 1 cm lateral to MCL	Short	Long MDM	Early peaking ESM grade IV	Atrial fibrillation	S3	Loud	11.4
Patient 2	DOE, NYHA functional class III, easily fatigued	Fifth IC, 0.5 cm lateral to MCL	Short	Long MDM	ESM grade IV	NSR	None	Loud	12.8
Patient 3	Asymptomatic	Fifth IC, medial to MCL	Long	Short MDM, PSM grade II	ESM grade III, EDM	Atrial fibrillation	None	Normal	13.4

DOE = dyspnea on exertion; EDM = early diastolic murmur; ESM = ejection systolic murmur; IC = intercostal space; MCL = midclavicular line; MDM = mid-diastolic murmur; NSR = normal sinus rhythm; PSM = pan-systolic murmur.

**Table 2:** Echocardiographic Parameters at Baseline and Immediately After PTMC

Case	LVEF (%)	Echocardiographic Parameters Before PTMC										Echocardiographic Parameters After PTMC									
		IVSd (mm)	LVPWd (mm)	LVIDd (mm)	LVIDs (mm)	LA (mm)	MVA by Planimetry (cm^2^)	MG Across MV (mm Hg)	RVSP (mm Hg)	AVA by planimetry (cm^2^)	MG Across AV (mm Hg)	IVSd (mm)	LVPWd (mm)	LVIDd (mm)	LVIDs (mm)	LA (mm)	MVA by Planimetry (cm^2^)	MG Across MV (mm Hg)	RVSP (mm Hg)	AVA by Planimetry (cm^2^)	MG Across AV (mm Hg)
Case 1	50	12	12	49	36	63	0.8	25 (78)	79	0.7	54	12	12	49	36	63	1.5	07 (75)	35	0.7	55
Case 2	60	9 (z-score: +2.84)	9 (z-score: +1.99)	46	32	42	0.8	31 (69)	46	0.9 cm^2^ (0.78 cm^2^/m^2^ BSA)	49	9	9	46	32	42	1.5	6 (80)	30	0.9	54
Case 3	60	Patient was assessed after PTMC										11	11	49	35	47	1.6	05 (68)	29	1.3	47

AV = aortic valve; IVSd = interventricular septum thickness at end-diastole; LA = left atrium; LVEF = left ventricular ejection fraction; LVIDd = left ventricular internal diameter in diastole; LVIDs = left ventricular internal diameter in systole; LVPWd = left ventricular posterior wall thickness in diastole; MG = mean gradient; MV = mitral valve; RVSP = right ventricular systolic pressure; PTMC = percutaneous transvenous mitral commissurotomy.

**Table 3:** Hemodynamic Parameters at Baseline and After PTMC

Case	Hemodynamic Parameters Before PTMC						Hemodynamic Parameters After PTMC					
	Left Atrium Pressure (mm Hg)	LA-LV Gradient (mm Hg)	Mean Pulmonary Artery Pressure (mm Hg)	Aortic Pressure (mm Hg)	Aortic Regurgitation (Sellers Grade)	Peak-to-Peak Aorta-LV Gradient (mm Hg)	Left Atrium Pressure (mm Hg)	LA-LV Gradient (mm Hg)	Mean Pulmonary Artery Pressure (mm Hg)	Aortic Pressure (mm Hg)	Aortic Regurgitation (Sellers Grade)	Peak-to-Peak Aorta-LV Gradient (mm Hg)
Case 1	39	19	67	123/64 (on inotropes)	I	43	26	8.3	45	103/71 (off inotropes)	II	48
Case 2	35	21	46	77/60	II	38	21	7.4	30	90/57	II	39
Case 3	Patient was assessed after PTMC						21	8	16	111/47	II	41

LA = left atrium; LV = left ventricle; PTMC = percutaneous transvenous mitral commissurotomy.

**Figure 3:**
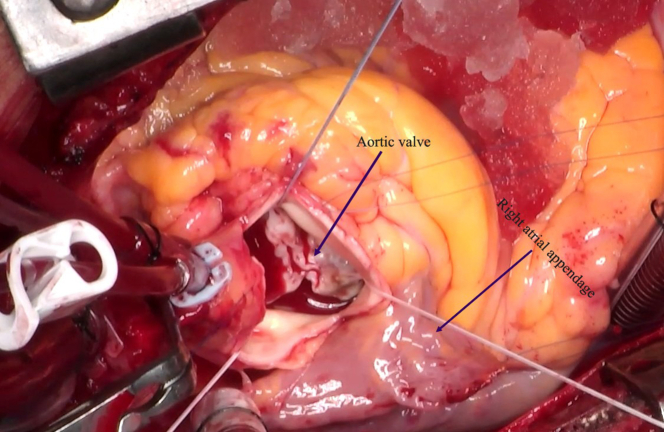
Intraoperative Image Intraoperative image of aortic valve replacement using a bileaflet mechanical valve secured with Ethibond 2-0 (17 mm) pledgeted sutures (Ethicon).

A few days after the procedure, the cardiothoracic vascular surgery team performed an aortic valve replacement (AVR) (Figure 3). At the 6 month follow-up, echocardiography revealed a normally functioning prosthetic aortic valve.

## Case 2

**Figure 4:**
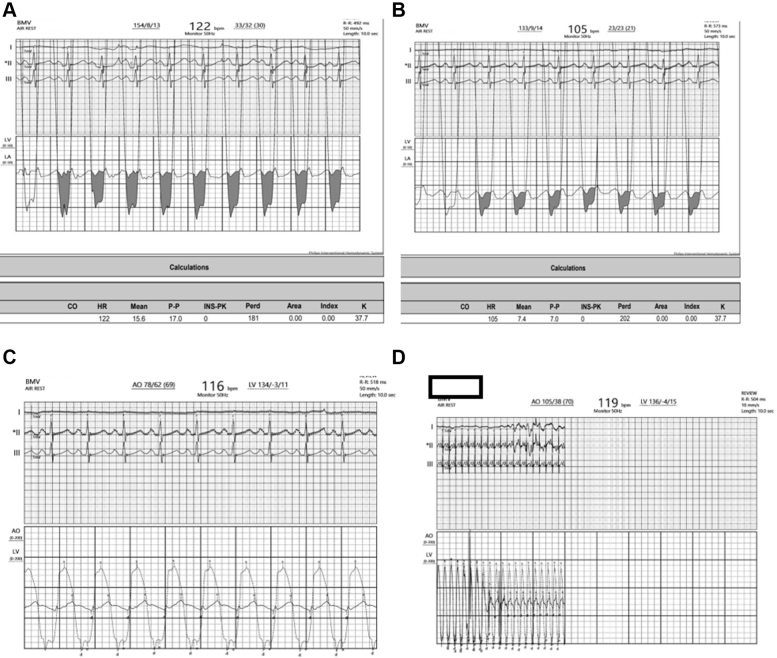
Hemodynamic Tracings Hemodynamic tracings showing the gradient across the mitral valve (A) before and (B) after percutaneous transvenous mitral commissurotomy. (C) Simultaneous (aorta–left ventricle) gradient after percutaneous transvenous mitral commissurotomy. (D) Pullback tracings from the left ventricle to the aorta with pigtail catheter.

A 14-year-old young man presented with progressive exertional dyspnea associated with palpitations, which he had experienced for 3 years. Two-dimensional echocardiography revealed features of RHS, with severe mitral stenosis (mitral valve area: 0.6 cm^2^ by planimetry) and severe aortic stenosis (peak/mean gradients across the aortic valve: 72/48 mm Hg) (Video 3). PTMC was performed with an aortogram that showed grade 2+ regurgitation; surprisingly, the aortic valve was crossed directly with the 5-F pigtail catheter without wire support, and gradients were taken across the aortic valve (Figure 4). The tracings revealed a reduced left ventricle-left atrial gradient post PTMC (Figures 4A and 4B), while both simultaneous left ventricle-aorta pull-back and simultaneous gradients were calculated (Figures 4C and 4D). The difference in pressure tracings gave an idea of the degree of aortic stenosis. The catheter tracings suggested that the increased gradients might be confounded owing to tachycardia and aortic regurgitation (Table 3). AVR was deferred, with a plan for close follow-up.

## Case 3

**Figure 5:**
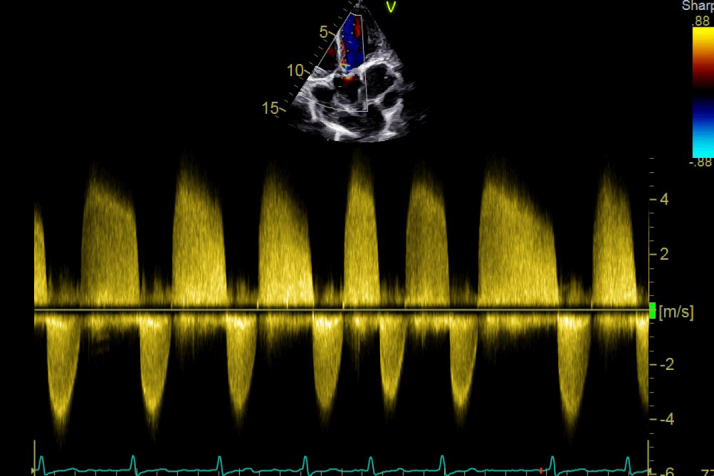
2D-TTE Continuous-wave Doppler tracing across the aortic valve showing a peak velocity of 4.1 m/s. s/o = severe aortic stenosis.

**Figure 6:**
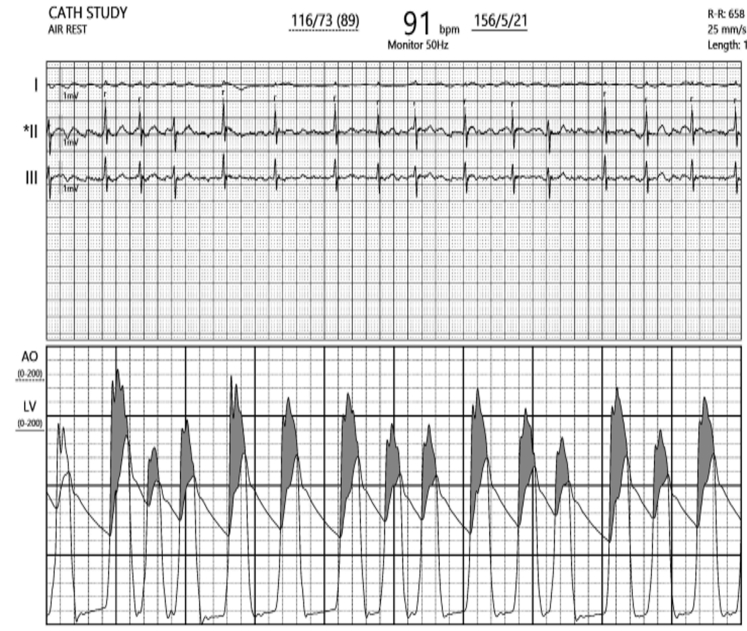
Hemodynamic Tracings Hemodynamic tracing showing the gradient across the aortic valve after balloon mitral valvotomy.

A 45-year-old woman diagnosed with RHD with moderate aortic stenosis and severe mitral stenosis 10 years prior had undergone PTMC at that time and remained relatively asymptomatic thereafter. However, echocardiogram during her follow-up showed severe aortic stenosis. Her aortic valve peak velocity was 4.1 m/s, but her aortic valve area was 1.2 cm^2^ (Figure 5, Video 4). A cardiac catheterization study was performed owing to discordant clinical and echocardiographic findings, and results suggested moderate aortic stenosis with falsely increased gradients due to significant aortic regurgitation (Sellers grade 2+) (Figure 6). The hemodynamic tracing in Figure 6 shows simultaneous left ventricular and ascending aortic pressure tracing, thus delineating all the gradients on a beat-to-beat basis. During an exercise stress test, the patient achieved 9 METs (metabolic equivalents of task), which was more than her sex- and age-matched predictions with normal blood pressure response, and she remained asymptomatic throughout the exercise testing. She is currently being planned for close follow-up.

## Discussion

### Case 1

Because both the valves were severely involved in this patient, with the mitral valve being thickened but pliable and suitable for PTMC, after due consultation with the cardiothoracic and vascular surgery teams and with the hemodynamic instability of the patient in mind, we performed a combined PTMC followed by an AVR in the same setting. Overall, the hemodynamic gradients with left ventricular hypertrophy and a mildly reduced ejection fraction supported our management strategy. The patient’s symptoms improved over the follow-up period.

### Case 2

Patient 2 was also intended for PTMC followed by AVR. But during PTMC, a 5-F pigtail catheter was found to easily cross the aortic valve without wire support, proving the lesion was not yet very tight, and retrospective evaluation of the echocardiographic measurements with the cinefluoroscopic assessment of concomitant aortic regurgitation revealed that the aortic stenosis was moderate after adjusting for body surface area, tachycardia, and aortic regurgitation. Adding to these factors, given a small aortic annulus (14 mm) and increased risk of patient prosthetic mismatch in prosthetic aortic valves <21 mm, was the possibility of repeated explorations after valve degeneration. Considering these issues, we decided to maintain a close follow-up on the aortic valve area and gradients rather than pursue an immediate surgical approach.

### Case 3

In the third patient, PTMC had been performed 10 years before when she was symptomatic. The aortic lesion was moderate at that time and had progressed slowly over the years. Although the patient was currently asymptomatic, a color Doppler assessment of the aortic valve was suggestive of severe aortic stenosis. However, the valve area determined by planimetry indicated that the valve was not severely stenosed. An invasive hemodynamic study confirmed that the gradient was due to significant aortic regurgitation, and a treadmill test assessment helped to rule out any hidden symptoms. Thus, conservative management was planned for her aortic valve.

Concomitant severe rheumatic aortic and mitral stenosis, being a double stenotic lesion, takes a serious toll on the quality of life of a patient by affecting cardiac output. The hemodynamic interactions between aortic stenosis in mixed/combined valvular diseases depend on the specific combination of the valvular lesions and the severity of the lesions, and they may result in diagnostic pitfalls at echocardiographic assessment, as most of the parameters are validated for single-valve diseases. Though the treatment of choice for such a multivalvular disease is not well defined, a double valve replacement (DVR) is generally considered the consensus in the developed world according to the 2020 American College of Cardiology/American Heart Association Valvular Heart Disease Guidelines and the 2017 European Society of Cardiology/European Association for Cardio-Thoracic Surgery Guidelines. However, a DVR has a more inferior prognosis than a single valve replacement, with twice the operative mortality.^4^ In one series, only one-third of patients who underwent DVR survived until age 15 years compared with half of the patients who underwent mitral valve repair and AVR.^5^ DVR is associated with higher intraoperative mortality, ranging between 5% and 12% in various series. Post–cardiopulmonary bypass myocardial dysfunction and low cardiac output syndrome were the most common causes of death in the immediate postoperative period, with other causes being sepsis, bleeding, arrhythmias, and, rarely, atrioventricular groove disruption.^6^ The practical logistics of maintaining the stricter international normalized ratio range (2.5-3.5) for DVR vs AVR (2.0-3.0) in low- and middle-income countries, in resource-limited settings with poor access to health care and less patient health education, while also avoiding bleeding complications, make a DVR the less favorable choice. Another approach to this problem would be open mitral valve repair with AVR, however this protocol is limited by prolonged cardiopulmonary bypass time and the relative inexperience of current surgeons in mitral valve repair techniques. One study showed no statistically significant difference between the hospital mortality of a DVR and a mitral repair and AVR.^7^ Conversely, although PTMC is frequently the intervention of choice for severe rheumatic mitral stenosis, the same cannot be said about severe rheumatic aortic stenosis. It is often considered an inferior choice compared to AVR, as it does not improve the prognosis significantly.^8^ Overall, aortic regurgitation is commonly associated with rheumatic aortic stenosis, thus making balloon aortic valvotomy contraindicated. This is why various percutaneous treatment modalities have been attempted, with the most recent one being a simultaneous PTMC with transcatheter AVR.^9^ Such a procedure is often limited in low- and middle-income countries owing to the costs involved, especially to the socioeconomic strata bearing the brunt of multivalvular RHD.

In this scenario, we propose a combination of PTMC followed by AVR as a management alternative for concomitant aortic and mitral stenosis. There is a dearth of literature highlighting this issue. An added advantage of this protocol is that PTMC often increases the blood flow to the aortic valve by one-third and thus may help unmask any trace aortic regurgitation that might have been missed.^10^ This procedure provides the advantage of a single valve replacement, with relatively more affordability in the current setup. In our first patient, PTMC followed by AVR was considered the best therapeutic option after a thorough discussion with the heart team, providing reassurance in our decision-making process.

## Funding Support and Author Disclosures

The authors have reported that they have no relationships relevant to the contents of this paper to disclose.

## Appendix

Video 1 Parasternal Short-Axis View Showing Thickened Rheumatic Mitral Valves Parasternal short-axis view showing thickened rheumatic mitral valves.

Video 2 Cinefluoroscopic View Showing the Disappearance of the Waist of the Inoue Balloon on Inflation, Signifying a Successful Mitral Valvotomy Cinefluoroscopic view showing the disappearance of the waist of the Inoue balloon, signifying a successful mitral valvotomy.

Video 3 Parasternal Long Axis View Showing Turbulent Color Flow Across the Thickened Aortic Valve and the Mitral Valve With Significant Aortic Regurgitation Parasternal long-axis view showing turbulent color flow across the aortic valve and laminar flow across the mitral valve.

Video 4 Parasternal Short-Axis View of a Rheumatic Thickened Aortic Valve Parasternal short-axis view of a rheumatic thickened aortic valve.
